# A Practical Treatise on Medical Diagnosis, for Students and Physicians

**Published:** 1894-05

**Authors:** 


### Book Reviews.

A Practical Treatise on Medical Diagnosis for Students and Physicians. By John H. Musser, M.D., Assistant Professor of Clinical Medicine in the University of Pennsylvania; Physician to the Philadelphia and the Presbyterian Hospital; Consulting Physician to the Woman's Hospital of Philadelphia and to the West Philadelphia Hospital for Women; Fellow of the College of Physicians of Philadelphia; President of the Pathological Society of Philadelphia, etc., etc. Illustrated with one hundred and sixty-two wood cuts and two colored plates. Philadelphia: Lea Bros. & Co., 1894.

This treatise on medical diagnosis is the most complete and satisfactory we have yet seen. Besides being well written and splendidly arranged, it contains all that clinical facts and recent scientific investigations have proven of incontestible value. Dr. Musser truly remarks in his preface "that the necessity for elaborate descriptions or extended lists of minutiæ as guides to differentiation is being rapidly displaced by the use of instruments of precision." Accordingly, in his book superfluous detail gives preference to the advanced methods as now used by the most skilful and scientific diagnosticians. In reading his book we can but be impressed with the rapid progress which has been made in this direction in recent years. The skilful and conscientious diagnostician of to-day must be a man of no mean ability, but equipped with a thorough knowledge of bacteriology, pathology, microscopy and symptomatology.

For the "practitioner as a reliable, practical guide to diagnosis in his daily work," as well as for the student as an aid in laboratory research and its application to disease, etc., the work is invaluable. The chapter on Morbid Processes and their Symptomatology, arranged especially for students, is clear and instructive. He divides the morbid processes into three: First, alterations in the blood and circulation; second, disturbances of nutrition; third, anomalies of growth. The author says the *object of diagnosis* is to determine the condition of the living patient who may be suffering from disease. It implies not only that the phenomena of disease are detected, but also that the effects of the disease on the organism are determined, and that the morbid process, which is the *cause* of the phenomena, is ascertained. Even this is too restricted an idea of diagnosis. It should include also the recognition of the cause of the morbid process. The latter is known as the *ætiological diagnosis.* With this broad and correct view of diagnosis, the author has not confined himself to symptoms, but goes deeply into etiology, heredity, environments, temperaments, etc., a knowledge of each of which is so essential to the diagnostician and to the employment of rational therapeutics.

The fluency of this gifted writer renders the reading pleasant. His acknowledged ability as a physician and investigator gives confidence in all he says. V. H. T.

